# Real-world impact of latanoprostene bunod ophthalmic solution 0.024% in glaucoma therapy: a narrative review

**DOI:** 10.3389/fopht.2025.1554777

**Published:** 2025-03-28

**Authors:** W. Daniel Stamer, Thomas Chiu, Da-Wen Lu, Tsing Hong Wang, Prin Rojanapongpun, Ngamkae Ruangvaravate, Youn Hye Jo, Marlene R. Moster, Murray Fingeret, Nora Lee Cothran, Jessica Steen, Ian Benjamin Gaddie, Ömür Uçakhan-Gündüz, Wesam Shamseldin Shalaby, Cindy M. L. Hutnik

**Affiliations:** ^1^ Department of Ophthalmology, School of Medicine, Duke University, Durham, NC, United States; ^2^ The Chinese University of Hong Kong, Hong Kong Eye Hospital, Hong Kong, Hong Kong SAR, China; ^3^ Department of Ophthalmology, Tri-Service General Hospital, National Defense Medical Center, Taipei, Taiwan; ^4^ Department of Ophthalmology, National Taiwan University Hospital, Taipei, Taiwan; ^5^ Chulalongkorn University & King Chulalongkorn Memorial Hospital, Bangkok, Thailand; ^6^ Siriraj Hospital, Mahidol University, Bangkok, Thailand; ^7^ Department of Ophthalmology, Seoul Konkuk University Hospital, Seoul, Republic of Korea; ^8^ Sidney Kimmel Medical College, Thomas Jefferson University, Philadelphia, PA, United States; ^9^ Department of Ophthalmology, Wills Eye Hospital, Philadelphia, PA, United States; ^10^ State University of New York, College of Optometry, New York, NY, United States; ^11^ Department of Glaucoma, The Eye Institute of West Florida, Largo, FL, United States; ^12^ Nova Southeastern University College of Optometry, Fort Lauderdale, FL, United States; ^13^ Gaddie Eye Centers, Louisville, KY, United States; ^14^ Ankara University School of Medicine, Ankara, Türkiye; ^15^ Tanta Medical School, Tanta University, Tanta, Gharbia, Egypt; ^16^ Ivey Eye Institute, Western University, London, ON, Canada

**Keywords:** glaucoma, open-angle, intraocular pressure, nitric oxide donors, ocular hypertension, prostaglandin analog

## Abstract

Latanoprostene bunod ophthalmic solution (LBN) 0.024% is a topical nitric oxide (NO)-donating prostaglandin F2α (PGF2α) analog first approved in November 2017 for reduction of intraocular pressure (IOP) in patients with ocular hypertension (OHT) or open-angle glaucoma (OAG). This narrative review describes the unique mechanism of action of LBN and summarizes available real-world data. Upon instillation, LBN is metabolized into latanoprost acid and butanediol mononitrate, which is further reduced to NO and an inactive metabolite. Latanoprost acid increases aqueous humor outflow primarily through the uveoscleral (unconventional) pathway, whereas NO increases outflow through the trabecular (conventional) pathway. Eight studies were identified: 2 studies in newly diagnosed, treatment-naïve patients with OHT or OAG, 4 studies of adjunctive therapy in patients with glaucoma receiving other IOP-lowering therapies, and 2 studies in which patients with glaucoma switched to LBN monotherapy or adjunctive therapy. Decreases in IOP after initiating LBN in newly diagnosed patients or adding/switching to LBN were generally consistent with reductions observed in clinical trials and sustained throughout the studies. Rates of discontinuation due to inadequate IOP lowering ranged from 12.2% to 17.1%. LBN was generally well tolerated in real-world studies; the most common adverse events were consistent with the known safety profile of LBN. Data from real-world studies provide important insights regarding the potential effectiveness and tolerability of LBN in the clinical setting and suggest that LBN is well tolerated and associated with significant, clinically meaningful, and durable reductions in IOP.

## 1 Introduction

Pharmacotherapy for intraocular pressure (IOP) lowering is the most common first-line intervention for open-angle glaucoma (OAG) (1–3). Topical prostaglandin analogs (PGAs) are often selected as initial therapy based on established efficacy and tolerability and a convenient dosing regimen, requiring only 1 drop per day (1–3). Alternative medications include topical beta-adrenergic antagonists, alpha_2_-adrenergic agonists, parasympathomimetics, rho-kinase inhibitors, and topical and oral carbonic anhydrase inhibitors (2). Patients may require a combination of IOP-lowering agents from different classes to adequately control IOP (1–3).

Latanoprostene bunod ophthalmic solution (LBN) 0.024% (heretofore referred to as LBN), the first topical nitric oxide (NO)-donating prostaglandin F2α (PGF2α) analog, was developed to provide greater and more efficient IOP lowering by combining the complementary mechanisms of action of PGF2α and NO into a single drug (4–6). A phase 1 study (KRONUS) in healthy Japanese male volunteers demonstrated significant reductions from baseline in 24-hour IOP over 14 days of treatment with LBN, supporting a potential benefit in OAG even with normal IOP (7). In the phase 2 VOYAGER study, once-daily LBN 0.024% provided significantly greater diurnal IOP lowering than once-daily latanoprost 0.005% over 28 days in patients with ocular hypertension (OHT) or OAG and achieved diurnal IOP ≤18 mm Hg in a significantly higher proportion of patients versus latanoprost at all study visits (8).

Compared to twice-daily timolol maleate 0.5%, once-daily LBN administered to patients with OHT or OAG significantly reduced nocturnal IOP and increased nocturnal ocular perfusion pressure (phase 2 CONSTELLATION study) (9), consistently produced significantly greater reductions in mean diurnal IOP over 3 months of treatment (phase 3 LUNAR (10) and APOLLO (11) studies), and was associated with a significantly higher proportion of patients achieving an IOP reduction of ≥25% at all time points assessed (LUNAR and APOLLO). Open-label extensions of APOLLO and LUNAR (12) and a single-arm, open-label study of LBN in Japanese patients with OHT or OAG (JUPITER) (6) demonstrated sustained IOP-lowering effects and a safety profile consistent with that of PGAs over 1 year of treatment. LBN was initially approved in the United States (US) in November 2017 for the reduction of IOP in patients with OHT and/or OAG (5, 13).

This narrative review describes the unique mechanism of action of LBN and summarizes real-world data from postmarketing studies to gain insight into the potential effectiveness and tolerability of LBN in the clinical setting.

## 2 Mechanism of action

**Figure 1:**
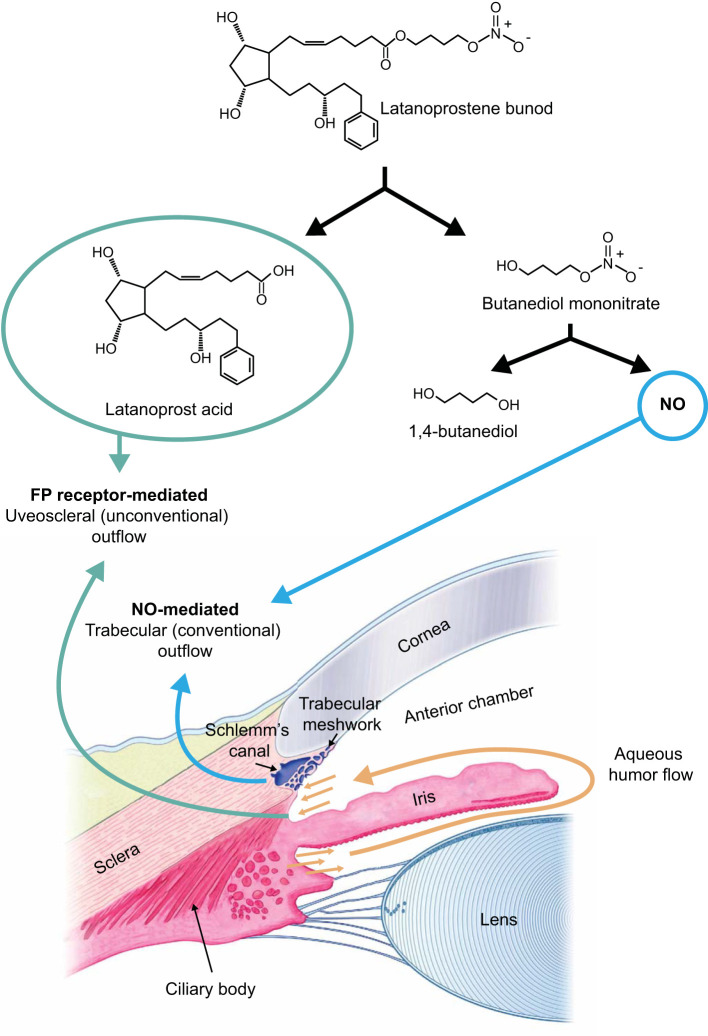
After being instilled into the eye, latanoprostene bunod (LBN) is rapidly metabolized by carboxyl ester hydrolysis into the prostaglandin F2α analog latanoprost acid (the active component of latanoprost) and butanediol mononitrate (6, 13, 16, 17). Subsequently, butanediol mononitrate is reduced to nitric oxide (NO) and an inactive metabolite, 1,4-butanediol. Binding of latanoprost acid to the F2α receptor leads to remodeling of the extracellular matrices in the ciliary body, increasing aqueous humor outflow through the uveoscleral (unconventional outflow) pathway (18–22). NO causes relaxation of the trabecular meshwork and may also increase permeability of Schlemm’s canal, thus increasing aqueous humor outflow via the trabecular meshwork (conventional outflow pathway) (8, 17, 39, 40). Figure adapted with permission from Kawase K, et al. Adv Ther. 2016;33:1612-1627, under a CC BY-NC license; Weinreb RN, et al. *US Ophthalmic Rev*. 2016;9(2):80-87, under a CC BY-NC license; and Kaufman PL, et al. Cholinergics. In: Sears ML, ed. *Pharmacology of the Eye: Handbook of Experimental Pharmacology*. Berlin: Springer-Verlag; 1984, with kind permission of Springer Nature (6, 16, 41).

Aqueous humor is secreted by the ciliary processes and drains via 2 independent pathways: the trabecular meshwork/Schlemm’s canal (the conventional outflow pathway, which accounts for 70% to 90% of outflow) and the uveoscleral pathway (the unconventional outflow pathway; **Figure 1**) (14–16). The balance between aqueous humor secretion and drainage determines IOP (14). Open-angle glaucoma is associated with increased resistance to aqueous drainage via the trabecular meshwork, whereas the iris typically obstructs access to the drainage pathways in angle-closure glaucoma (14). The resulting increase in IOP places mechanical stress and strain on the posterior structures of the eye, leading to optic nerve fiber dysfunction and death of retinal ganglion cells, followed by progressive visual field loss (14, 17).

LBN reduces IOP through 2 distinct mechanisms by targeting both the trabecular and the uveoscleral outflow pathways (**Figure 1**). Metabolism of LBN following topical instillation results in the generation of 2 active compounds: latanoprost acid and NO (6, 13, 17). Latanoprost acid binds to and activates prostaglandin FP receptors in the ciliary muscle, leading over time to remodeling of the extracellular matrices in the ciliary body (18–22). This remodeling enlarges interstitial spaces within the longitudinal ciliary muscle bundles and increases aqueous humor outflow through the uveoscleral pathway. The initial reduction in IOP from topical latanoprost acid may also be due to ciliary muscle relaxation (19, 21). Additionally, multiple studies show that prostaglandins, including latanoprost acid, increase aqueous humor outflow, in part, through the trabecular network (21–25).

The second mechanism by which LBN lowers IOP is by targeting the trabecular meshwork with NO (**Figure 1**). Cells in Schlemm’s canal produce NO, which regulates IOP by remodeling the extracellular matrix within the trabecular meshwork, thus increasing aqueous humor outflow (17, 26, 27). The trabecular meshwork has contractile properties similar to those of smooth muscle (26–31). Rho kinase activity and intracellular calcium signaling mediate trabecular meshwork contraction (27, 30, 32). In normal eyes, NO activates the soluble guanylate cyclase and cyclic guanosine monophosphate (cGMP) cascade, leading to inhibition of both Rho kinase activity and intracellular calcium signaling, thus resulting in relaxation of the trabecular meshwork (17, 27, 29, 30, 32, 33).

Deficient NO signaling is thought to contribute to chronic contraction and subsequent extracellular matrix remodeling of the trabecular meshwork and elevated IOP in glaucoma. The eyes of patients with OAG have decreased levels of NO markers compared with normal eyes, which is interpreted as reflecting a deficiency in NO signaling (33–36). The NO released by LBN acts in the same way as endogenous NO, increasing aqueous humor outflow through the trabecular pathway and decreasing IOP (**Figure 1**) (6, 8, 16, 17, 33, 37–41). Accumulating data suggest that NO released from LBN may also confer neuroprotective benefits via effects on optic nerve head blood flow and macular retinal blood vessel density (42, 43).

## 3 Real-world studies

A literature review identified 8 real-world studies of LBN, including 2 studies in newly diagnosed, treatment-naïve patients with OHT or OAG (44, 45), 4 studies of adjunctive therapy in patients with glaucoma receiving other IOP-lowering therapies (46–49), and 2 studies in which patients with glaucoma switched to LBN monotherapy or adjunctive therapy (50, 51) (**Table 1**). With the exception of 1 study (50), all real-world studies were retrospective.

**Table 1:** Real-world studies of latanoprostene bunod 0.024%.

Study, Population, and Design	Sample Size	Medication(s)	FU Duration	Baseline IOP	Key Efficacy Findings	Key Safety Findings
Monotherapy in newly diagnosed patients with OHT or OAG						
Okeke et al., 2020 (44) • Retrospective, multicenter (United States) medical chart review • Pts ages ≥18 y with newly diagnosed OHT or OAG prescribed LBN as their initial IOP-lowering medication who had ≥2 FU visits	N=65*	LBN 0.024% QD	Visit 1: 43 (41) days after starting LBN Visit 2: 141 (76) days after starting LBN	Study eye 21.7 (5.9)	• IOP in study eye decreased from BL to 14.7 (4.1) at visit 1 and 14.4 (3.2) at visit 2 • RFB in IOP was 7.1 (4.7) (30.8%) at visit 1 and 7.3 (5.1) (30.8%) at visit 2 (both P<0.0001) • Among pts with BL IOP >21: RFB was 10.0 (4.5) (37.1%) at visit 1 and 11.1 (4.6) (40.9%) at visit 2 (both P<0.0001) • ≥20% RFB in IOP (visit 1/visit 2): 79.4%/78.5% • ≥30% RFB in IOP (visit 1/visit 2): 54.0%/50.8% • ≥40% RFB in IOP (visit 1/visit 2): 27.0%/28.1%	• No systemic AEs • ≥1 ocular AE in 33 pts (50.7%) • Most common AEs: blurred vision (n=10, 12 events), dryness (n=8, 11 events), itching (n=5, 6 events), irritation (n=5, 6 events), and light sensitivity (n=5, 5 events) • 1 pt experienced ocular redness • No discontinuations due to AEs • No meaningful changes in VA or CDR measurements
Wang et al., 2020 (45) • Retrospective, multicenter (China) cohort study • Pts newly diagnosed with OHT or primary OAG and IOP ≥21 mm Hg	N=313	Latanoprost 0.005% QD (n=104) LBN 0.024% QD (n=94) Timolol 0.5% BID (n=115)	3 mos	Latanoprost: 24.1 (1.1) LBN: 24.0 (1.2) Timolol: 24.2 (1.7)	• IOP significantly decreased from BL at 3 mos: Latanoprost: 19.5 (1.0; P<0.0001), LBN: 17.5 (1.9; P<0.0001), and timolol: 19.7 (1.1; P<0.0001) • RFB in IOP was significantly greater with LBN vs latanoprost (P<0.0001) or timolol (P<0.0001)	• Overall incidence of ocular AEs was higher with latanoprost (16%) and LBN (14%) vs timolol (6%, P=0.031) • Most common AEs with latanoprost/LBN: eye irritation (4%/4%), conjunctival hyperemia (4%/3%), and eye pain (3%/3%) • At 3 mos, significant decreases from BL in heart rate, diastolic BP, and systolic BP observed with timolol but not with latanoprost or LBN • Mean % changes in corneal thickness were similar with latanoprost (1.2%), LBN (1.2%), and timolol (1.1%)
Adjunctive therapy in patients with inadequately controlled glaucoma						
Radell et al., 2021 (46) • Retrospective, single-center (United States) electronic medical records review • Pts with mild-to-severe glaucoma who did not achieve adequate IOP control with other agents or with laser or surgical treatments and were prescribed LBN	N=56 (102 eyes)	LBN 0.024% QD (32 switched from latanoprost, 9 from bimatoprost, 9 from travoprost, 3 from tafluprost, and 3 from fixed-dose combination of latanoprost/netarsudil)^†^	Mean FU duration of 7.9 mos Mean (SD) time to FU • First FU visit: 38.7 (36.5) days • Final FU visit: 235.9 (160.8) days	16.2 (4.3)	• IOP decreased from BL to 14.0 (3.6) at ≥7 days after starting LBN (IOP decrease of 2.1 [3.5]; P<0.0001) and to 13.7 (3.8) at most recent visit during which the patient was still taking LBN (mean [SD], 235.9 [160.8] days; IOP decrease of 2.5 [3.3]; P<0.0001)) • IOP decreased by ≥2 in 60% of eyes, by ≥3 in 46% of eyes, and by ≥4 in 34% of eyes • 7 pts (12.5%) discontinued LBN because of inadequate IOP control	• AEs reported in 8 pts (14.3%), including pain, itching, throbbing, and discomfort • 4 pts discontinued LBN because of AEs
Zhou et al., 2022 (47) • Retrospective, single-center (United States) electronic medical records review • Pts with glaucoma using ≥3 topical IOP-lowering agents who initiated adjunctive therapy with LBN 0.024%^‡^	N=33 (53 eyes)	LBN 0.024% QD	12 mos	19.9 (6.0)	• IOP decreased from BL to 17.3 (5.5) at 3 mos (RFB, 2.6 [6.6], 9%, P<0.01) to 17.2 (4.7) at 6 mos (RFB, 3.6 [7.4], 11%, P<0.01) and 16.0 (4.5) at 12 mos (RFB, 5.8 [7.4], 19%, P<0.01) • ≥20% RFB in IOP (mos 3/6/12): 35%/40%/57% • ≥30% RFB in IOP (mos 3/6/12): 22%/29%/39% • ≥40% RFB in IOP (mos 3/6/12): 10%/17%/25% • 7 eyes (13%) remained refractory to treatment and required surgery	• No discontinuations due to AEs
Mehta et al., 2022 (48) • Retrospective, multicenter (United States) cohort study • Pts with glaucoma already receiving other topical agents (≤4) who initiated adjunctive therapy with LBN 0.024% or netarsudil 0.02%	N=136 (136 eyes)	LBN 0.024% QD (n=41 pts/eyes)^‡^ Netarsudil 0.02% QD (n=95 pts/eyes)	Mean (SD)/range treatment duration: LBN: 79.4 (51.8) days/14–210 days Netarsudil: 53.7 (28.5) days/5–120 days	LBN: 19.4 (5.7) Netarsudil: 20.3 (6.1)	RFB in IOP: • LBN: 2.9 (3.7), 13.6%, P<0.0001 • Netarsudil: 3.9 (4.6), 17.5%, P<0.0001 • In both cohorts, RFB in IOP did not depend significantly on BL number of IOP medications • 7 pts (17.1%) discontinued LBN and 11 pts (11.6%) discontinued netarsudil because of inadequate IOP lowering	• 3 pts (3.2%) discontinued netarsudil because of erythema • No sight-threatening complications in any pt
Bahr et al., 2023 (49) • Retrospective, single-center (United States) cohort study with electronic medical records review • Pts with primary OAG on combination therapy with an alpha-agonist, a beta-blocker, a carbonic anhydrase inhibitor, and PGA who either added netarsudil 0.02% or substituted PGA with LBN 0.024%	N=35 (35 eyes)	LBN 0.024% QD (n=11 pts/eyes)^‡^ Netarsudil 0.02% QD (n=24 pts/eyes)	Mean (SD) time to FU: LBN: 59 (19) days Netarsudil: 48 (17) days	Significantly higher in netarsudil cohort (20.9 [5.1]) than LBN cohort (17.8 [3.4]; P=0.04)	• Significantly greater RFB in IOP in pts receiving netarsudil (4.0 [1.9], 17%) vs LBN (0.4 [1.8], 2%; *P*=0.007) • Netarsudil group was significantly more likely to achieve ≥10% RFB in IOP (71% vs 18%; P=0.009)	No significant changes in VA in either group
Switching (monotherapy or adjunctive therapy) in patients with OHT or OAG						
Zanutigh et al., 2023 (50) • Prospective, single-center (Argentina), nonrandomized, nonmasked, comparative case series • Pts >21 years of age with chronic OAG being treated for 6–12 mos with latanoprost 0.005% with no known intolerance	N=36 (72 eyes)	LBN 0.024% QD (started after 1-week washout period)	3 mos	13.4 (2.1)	• OSDI score improved from 17.8 (12.1) at BL (during latanoprost treatment) to 11.1 (10.5) at 3 mos after starting LBN (P<0.01) • OSDI score worsened in 8 pts (22.2%), remained unchanged in 1 (2.8%), and improved in 27 (75.0%) • 30.6% increase in percentage of pts with normal OSDI score (<13); from 41.6% at BL to 72.2% • Fluorescein staining (Oxford scale) improved from BL (0.6 [0.7]) to month 3 (0.2 [0.8]; P=0.01) • No changes in IOP from BL to 1 month (13.2 [2.0]) and 3 mos (13.1 [1.7]) after starting LBN • No changes in tear breakup time or Schirmer test scores	• No change in best-corrected VA throughout study
Okeke et al., 2024 (51) • Retrospective, multicenter (United States) chart review • Pts ≥18 years of age with mild-to-moderate OHT or OAG on 1–2 topical IOP-lowering medications^§^ who switched 1 or both to LBN and had ≥2 visits after starting LBN	N=49*	LBN 0.024% QD (prior to switch, 38 pts on monotherapy with PGA [n=32] or non-PGA [n=6] and 11 on dual therapy with PGA and non-PGA [n=9] or 2 non-PGA products [n=2]) After switch, 40 pts taking LBN monotherapy and 9 taking LBN + 1 other IOP-lowering agent^§^	Visit 1: median (IQR), 28 (21–41) days after starting LBN Visit 2: median (IQR), 126 (80–160) days after starting LBN	21.9 (4.2)	• Overall: IOP decreased from BL (prior to LBN) to 16.5 (3.7) at visit 1 (reduction, 5.4 [3.3]; P<0.001) and 15.9 (4.3) at visit 2 (reduction, 5.2 [4.8]; P<0.001); ~25% reduction at both visits • ≥2 RFB in IOP (visit 1/visit 2): 89.4%/85.0% • ≥3 RFB in IOP (visit 1/visit 2): 83.0%/75.0% • ≥4 RFB in IOP (visit 1/visit 2): 70.2%/67.5% • ≥5 RFB in IOP (visit 1/visit 2): 59.6%/60.0% • IOP lowering and responder analyses in pts using a PGA with or without other IOP-lowering product and in those using PGA monotherapy prior to switch from previous PGA to LBN were consistent with overall dataset • 6 pts (12.2%) discontinued LBN because of inadequate IOP lowering	• No systemic AEs • 14 ocular AEs in 9 pts (17.6%) • Only ocular AEs occurring in >1 patient were floaters (n=3, 6.1%) and eye dryness (n=2, 4.1%) • No discontinuations due to AEs • No significant changes in VA or CDR

Data for fluorescein staining, IOP, and OSDI values presented as mean (SD). IOP reported as mm Hg.

*Eye with the highest baseline IOP was the study eye. If baseline IOP was the same in both eyes, then the right eye was the study eye.

^†^Patients switched from a PGA or a PGA-containing fixed-dose combination but continued to take other glaucoma medication.

^‡^LBN was substituted for PGA in patients previously taking PGA.

^§^Combination product was considered 1 therapy.

AE, adverse event; BL, baseline; BP, blood pressure; CDR, cup-to-disk ratio; FU, follow-up; IOP, intraocular pressure; IQR, interquartile range; LBN, latanoprostene bunod; mos, months; OAG, open-angle glaucoma; OHT, ocular hypertension; OSDI, ocular surface disease index; PGA, prostaglandin analog; pt, patient; RFB, reduction from baseline; SD, standard deviation; VA, visual acuity.

### 3.1 Initial therapy

A retrospective, multicenter medical chart review assessed the effects of LBN 0.024% in 65 adults with newly diagnosed OHT or OAG who had at least 2 follow-up visits (spanning ≥2 months) (44). Initiation of LBN therapy was associated with a mean (SD) reduction from baseline (RFB) of 7.1 (4.7) mm Hg at follow-up visit 1 (~1.4 months) and 7.3 (5.1) mm Hg at visit 2 (~4.7 months); 30.8% reductions and P<0.0001 were seen at both visits. Mean percent reductions in IOP of 37.1% and 40.9% were observed in patients with baseline IOP of >21 mm Hg at visits 1 and 2, respectively. At both visits, more than half of patients had achieved ≥30% RFB in IOP. The most common adverse events (AEs) were blurred vision, dryness, itching, irritation, and light sensitivity. No meaningful changes in visual acuity (VA) or cup-to-disk ratio (CDR) were reported.

Wang and colleagues (45) conducted a retrospective, multicenter study comparing LBN QD (n=94) with latanoprost 0.005% QD (n=104) and timolol 0.5% BID (n=115) in patients newly diagnosed with OHT or OAG and with IOP of ≥21 mm Hg. In all 3 treatment cohorts, baseline IOP was approximately 24 mm Hg and decreased significantly (P<0.0001) from baseline to 3 months (mean [SD]: LBN, 17.5 [1.9]; latanoprost, 19.5 [1.0]; timolol, 19.7 [1.1]). The RFB in IOP was significantly greater with LBN compared with latanoprost or timolol (both P<0.0001). Latanoprost and LBN were associated with a higher incidence of AEs (16% and 14%, respectively; most commonly eye irritation, conjunctival hyperemia, and eye pain) than timolol (6%). However, timolol, but not latanoprost or LBN, was associated with significantly reduced heart rate and blood pressure.

### 3.2 Adjunctive therapy

A retrospective, single-center study followed 56 patients (102 eyes) with mild-to-severe glaucoma inadequately controlled with medical therapy or prior laser or surgical treatments who initiated LBN as a replacement for a PGA or for a fixed-dose combination of latanoprost/netarsudil (46). Patients took a mean of 3 glaucoma medications prior to starting LBN and throughout a median follow-up period of 7.9 months in the study, although non-PGA agents were not specified. The mean (SD) IOP was 16.2 (4.3) mm Hg at the visit during which LBN was prescribed. The mean (SD) RFB in IOP was 2.1 (3.5) mm Hg at the first follow-up visit (mean, 38.7 days) and 2.5 (3.3) mm Hg at the last visit (mean, 236 days; both P<0.0001). Clinically meaningful reductions of ≥2, ≥3, and ≥4 mm Hg were observed in 60%, 46%, and 34% of eyes, respectively. Seven patients (12.5%) discontinued LBN because of inadequate IOP control. Eight patients (14.3%) reported AEs, including pain, itching, throbbing, and discomfort; 4 patients discontinued LBN because of these AEs.

A retrospective, single-center study in 33 patients (53 eyes) evaluated LBN as adjunctive therapy in patients with severe glaucoma refractory to ≥3 topical IOP-lowering agents (47). LBN was substituted for PGA in patients previously taking a PGA. Mean (SD) IOP was 19.9 (6.0) mm Hg at baseline and, following initiation of LBN therapy, decreased by 2.6 (6.6) mm Hg (9%) at 3 months (49 eyes), 3.6 (7.4) mm Hg (11%) at 6 months (35 eyes), and 5.8 (7.4) mm Hg (19%) at 12 months (28 eyes; all P<0.01). At 12 months, RFB was ≥20% in 57% of eyes, ≥30% in 39%, and ≥40% in 25%. Surgery was required to control IOP in 7 of 53 eyes (13%). In this study, LBN was well tolerated, and no patients discontinued treatment because of AEs.

A retrospective multicenter cohort study assessed the effectiveness of LBN and the rho-kinase inhibitor netarsudil 0.02% as an adjuvant therapy in patients with glaucoma receiving up to 4 topical agents (48). The study included patients who had undergone prior surgical glaucoma procedures. Initiation of adjunctive therapy or replacement of the previous PGA with LBN (n=41 patients/eyes; mean [SD] baseline IOP, 19.4 [5.7] mm Hg; mean treatment duration, 79.4 days) was associated with a mean (SD) RFB in IOP of 2.9 (3.7) mm Hg (13.6%; P<0.0001) (48). Of the 95 patients (95 eyes) that underwent adjunctive therapy with netarsudil 0.02%, the mean (SD) IOP was 20.3 (6.1) mm Hg at baseline and decreased by 3.9 (4.6) mm Hg (17.5%; P<0.0001), which was comparable to the IOP-lowering effect of LBN. Changes in IOP did not correlate significantly with the baseline number of IOP medications. Netarsudil was discontinued in 11 patients (11.6%) for lack of effectiveness and 3 patients (3.2%) because of erythema. LBN was discontinued in 7 patients (17.1%) for lack of effectiveness.

A retrospective, single-center cohort study compared LBN (substituted for a PGA; n=11 patients/eyes; mean time to follow-up, 59 days) with netarsudil (added to the regimen; n=24 patients/eyes; mean time to follow-up, 48 days) in patients with primary OAG taking combination therapy with an alpha-agonist, a beta-blocker, a carbonic anhydrase inhibitor, and a PGA (49). This study excluded patients with prior glaucoma surgery. Mean (SD) baseline IOP was significantly higher in the netarsudil cohort (20.9 [5.1] mm Hg) than the LBN cohort (17.8 [3.4] mm Hg; P=0.04). Compared to LBN-treated patients, those receiving netarsudil had a significantly greater mean (SD) RFB in IOP (4.0 [1.9] vs 0.4 [1.8] mm Hg; P=0.007) and were significantly more likely to achieve ≥10% RFB in IOP (71% vs 18%; P=0.009). No significant changes in VA occurred in either cohort.

### 3.3 Switching

Okeke and colleagues (51) evaluated the effectiveness and safety of switching (primarily due to inadequate IOP lowering) to LBN in a retrospective chart review of 49 adults with mild-to-moderate OHT or OAG previously using 1 to 2 topical IOP-lowering medications. After switching, 40 patients were taking LBN monotherapy and 9 were taking LBN plus 1 other IOP-lowering therapy. The mean (SD) IOP before switching was 21.9 (4.2) mm Hg. Overall, IOP decreased by a mean (SD) of 5.4 (3.3) mm Hg to the first follow-up visit (median, ~1 month) and by 5.2 (4.8) mm Hg at the second follow-up visit (median, ~4 months; both visits, ~25%, P<0.001). At visit 2, the proportion of patients with an RFB in IOP of ≥2, ≥3, ≥4, and ≥5 mm Hg was 85.0%, 75.0%, 67.5%, and 60.0%, respectively. The magnitude of IOP lowering in subgroups of patients based on pre-switch treatment consisting of a PGA (PGA with or without another IOP-lowering product or PGA monotherapy) were consistent with those of the overall study population. Six patients (12.2%) discontinued LBN because of inadequate IOP lowering. Nine patients (17.6%) reported 14 ocular AEs, with only floaters and eye dryness occurring in more than 1 patient. There were no discontinuations due to AEs. There were no significant changes in VA or CDR.

A prospective, single-center, nonrandomized, nonmasked case series evaluated the ocular surface tolerance of LBN 0.024% compared with latanoprost 0.005% in patients with chronic OAG (50). Patients (N=36) who were previously treated for 6 to 12 months with latanoprost 0.005% with no known intolerance switched to LBN after a 1-week washout and were monitored for 3 months. After patients switched to LBN, a statistically significant improvement was observed in ocular surface disease index (OSDI) score and fluorescein staining/Oxford scale (ocular surface damage; both P≤0.01), but not in break-up time or Schirmer test results. The percentage of patients with a normal OSDI score (<13) increased from 41.6% during latanoprost treatment to 72.2% at 3 months after starting LBN (30.6% increase). Both best-corrected VA and IOP (which was low and well controlled at baseline; mean, 13.4 mm Hg) remained stable.

## 4 Discussion

Results of real-world studies showed reduction in IOP after initiating LBN in newly diagnosed patients (44, 45), as adjunctive therapy in patients receiving other IOP-lowering therapies (46–49), and after switching from IOP-lowering medications to LBN (51). The reductions in IOP observed in these studies were comparable to or higher than those observed in clinical trials (i.e., mean decrease of 3.9 mm Hg at 2 weeks, 3.1 mm Hg at 3 months, 2.1 mm Hg at 6 months, and 2.5 mm Hg at 12 months, based on a meta-analysis of pooled data from 9 trials (52)). Among studies reporting the percentages of patients with clinically meaningful reductions in IOP, rates of achieving RFB in IOP ≥2 mm Hg at the final visit ranged across 2 studies from 60% to 85% (46, 51), and rates of achieving RFB in IOP ≥20% at the final visit ranged across 2 studies from 57% to 78.5% (44, 47). Further, decreases in IOP were sustained over the course of the real-world studies, which ranged in mean duration from 3 to 4.7 months in studies of LBN as initial therapy (44, 45) and from 2 to 12 months in studies of LBN as adjunctive therapy (46–49); mean/median duration in the switch studies ranged from 3 to ~4 months (50, 51). Results of a study in patients with newly diagnosed OHT/OAG that observed a significantly greater RFB in IOP with LBN than with latanoprost or timolol (45) are consistent with those of clinical trials in which LBN demonstrated superior efficacy compared with latanoprost (8) or timolol (8, 10) and a network meta-analysis comparing the efficacy of LBN to other IOP-lowering therapies (53).

A single study (by Bahr and colleagues) reported only a modest reduction of IOP with LBN as adjunctive therapy (0.4 mm Hg, 2%) and a significantly greater reduction with netarsudil (4.0 mm Hg, 17%) (49). However, interpretation of this finding is limited by the significant difference in baseline IOP (17.8 mm Hg with LBN; 20.9 mm Hg with netarsudil) and the small number of LBN-treated patients (n=11) (49). This baseline difference is particularly important because higher baseline IOP has been reported to be associated with greater IOP reduction following both medications and surgical interventions for glaucoma (54). In contrast to a study by Bahr and colleagues, another multicenter study (by Mehta and colleagues) reported the comparable IOP-lowering effect of LBN and netarsudil when they were used as an adjuvant therapy in patients with glaucoma receiving up to 4 topical agents (48). Further larger prospective studies with LBN and netarsudil as adjunctive therapy will need to be undertaken to compare effectiveness of these drugs in lowering IOP.

Rates of discontinuation of LBN due to inadequate IOP lowering ranged in studies reporting this statistic from 12.2% to 17.1% (46, 48, 51). LBN was well tolerated, with generally low rates of individual AEs. The most common AEs were consistent with the known safety profile of LBN (i.e., ocular AEs such as instillation pain, eye irritation, and dryness) (13). Meaningful changes in VA and CDR were not observed (44, 49–51).

Interpretation of data from real-world studies examining the IOP-lowering effects of LBN is limited by several challenges inherent to real-world studies. These include lack of randomization, absence of placebo and/or active control groups, lack of masking, unknown treatment adherence rates, the possibility of inconsistent data collection leading to missing data, and associated bias in patient selection, data measurement/collection, and data assessment (44, 46, 55). Many of the studies included had small sample sizes, and all but one was retrospective in nature. Additionally, the requirement of ≥2 follow-up visits in some studies may have led to patient selection bias, as patients who discontinued LBN shortly after treatment initiation because of inadequate response or poor tolerability would have been excluded. Missing data from patients who discontinued LBN treatment because of inadequate response may have contributed to a higher RFB in IOP observed at later time points in some studies. Among studies in which patients were using multiple IOP-lowering medications, the specific combinations were generally not reported, which limits interpretation of the results. Short duration of most of the studies (6 of the 8) included may not have allowed evaluation of AEs related to PGA-associated periorbitopathy, which generally appear only after several months of treatment with PGA (56). Despite these limitations, data from real-world studies provide important insights regarding the potential effectiveness and tolerability of LBN in the clinical setting, especially considering the availability of only a single small clinical trial of LBN in treatment-naïve patients (9) and the lack of randomized clinical trials of adjunctive therapy with LBN and switching to LBN.

A prospective, single-center study demonstrated improvements in OSDI scores and fluorescein staining among 36 patients with chronic OAG who switched from latanoprost to LBN (50). The authors suggested that the observed improvement in ocular surface signs and symptoms after switching to LBN may, in part, be related to the ability of NO donors to promote secretion of proteins from acinar cells of the lacrimal gland (50, 57). Key limitations of this prospective switch study include its small sample size, short duration, and nonrandomized and open-label design (50).

### 4.1 Conclusions and future directions

LBN is a topical NO-donating PGA that offers significant IOP reduction by combining the complementary mechanisms of PGF2α and NO in a single formulation, enhancing both trabecular and uveoscleral outflow pathways. Data from real-world studies support that LBN is an important first-line agent that is well tolerated and can be used in conjunction with other glaucoma medications and surgical procedures. In addition to its potent IOP-lowering effects, NO may improve ocular blood flow and offer neuroprotective benefits, making LBN a valuable addition to glaucoma therapy.

Future research should evaluate the efficacy of LBN in diverse patient populations, particularly those with severe glaucoma or normal tension glaucoma with low presenting IOP. Studies involving these groups will help uncover the full therapeutic potential of LBN, particularly in complex and challenging cases. Additionally, a prospective, randomized comparative study between LBN and other PGAs in patients with OAG is warranted.

Despite the benefits of LBN, long-term use of glaucoma medications still presents challenges. Issues such as frequent dosing, ocular surface discomfort, and medication nonadherence remain barriers to effective treatment. Reducing the number of medications and minimizing AEs are key factors in improving patient adherence. LBN’s dual-action mechanism makes it a suitable candidate for combination therapy with aqueous suppressants. These combinations could integrate all 3 major pathways for reducing IOP—trabecular outflow, uveoscleral outflow, and aqueous production—into a single medication, a capability not seen in currently available fixed-combination therapies.

Sustained-release drug delivery systems have the potential to transform glaucoma management by maintaining consistent drug release and reducing fluctuations in IOP. This technology could reduce the burden of daily eye drops, improve treatment compliance, and provide more stable long-term control of IOP. The use of novel, slow-release NO donors and effective delivery platforms (e.g., use of nanoparticles) in treating ocular diseases is also being investigated for effective delivery of NO to the target region (58). Current FDA-approved PGA implants, which target uveoscleral outflow pathway, have already demonstrated the promise of sustained-release treatments (59–61). If LBN were developed in an implantable form, it could address multiple IOP-lowering pathways and potentially enhance treatment outcomes. Future research exploring fixed combination and sustained-release formulations of LBN could pave the way for more comprehensive and patient-friendly treatment strategies for glaucoma management.
